# Editorial

**Published:** 1897-06

**Authors:** 


### Editorial.

The invasion of the animal kingdom in the search for remedies gives most excellent promise of leading us back to the brave old days of yore when the basest excretions of the body were essential ingredients of cosmetics to beautify the complexions of the court ladies and were recommended as draughts to cool the blood of the *beau sabreurs* until late years, except in the absurd *materia medica* of the homeopaths, the mineral and vegetable kingdom supplied more than enough remedies to treat all the ills of humanity. But nowadays serum therapy has become fashionably prevalent. We are the fortunate possessors (or can easily become so) of a serum for almost every general disease. Could we but catch the wandering Jew and tap him for a little of his serum we might secure immunity from death itself.

The latest addition to the list of remedies of animal origin is an extract of the heads of leeches, which is intended to decrease the coagulability of the blood in those who are in need of a circulating fluid of less inconvenient density. We are informed of the merits of this extract in the annual report of a very dignified and reliable drug manufacturing establishment. According to the report it is an aqueous extract of the heads of sanguisuga medicinalis, hardened in alcohol, dried and pulverized. We are pleased to learn that it retards the coagulation of blood not only *in vitro*, but also in *corpore vivo*—it holds out no hope of retarding coagulation of blood in *corpore mortuo*. This effect is due to changes in the blood, microscopically demonstrable. Owing to the stimulating influence of the leech extract, the lencocytes are reanimated and with "higher vitality, mobilityand voraciousness" the return to the Homeric struggle with the invading hosts of pathogenic microbes. With the head of a sanguisuga medicinalis emblazoned on their banners they rush to the attack with all the address and abandon of those who swarmed up the breech of Ascalon. Are there any other extracts that are intended to fill a long felt want? If so, there is a yawning, yearning "want" in the graveyard awaiting them.

"Lord, is it I?"

The members of the medical profession of Atlanta, Ga., are much exercised over the impending trial, before a medical court, of thirteen of the best known physicians in the South on a charge of permitting their pictures to be printed in the newspapers.—*Ex.*

Who are they? Any one sending in a correct list of the thirte en will receive a highly embossed leather medal.

